# Delineation of Tumor Habitats based on Dynamic Contrast Enhanced MRI

**DOI:** 10.1038/s41598-017-09932-5

**Published:** 2017-08-29

**Authors:** Yu-Cherng Channing Chang, Ellen Ackerstaff, Yohann Tschudi, Bryan Jimenez, Warren Foltz, Carl Fisher, Lothar Lilge, HyungJoon Cho, Sean Carlin, Robert J. Gillies, Yoganand Balagurunathan, Raphael L. Yechieli, Ty Subhawong, Baris Turkbey, Alan Pollack, Radka Stoyanova

**Affiliations:** 1 University of Miami Miller School of Medicine, Miami, FL 33136 USA; 2 Department of Medical Physics, Memorial Sloan Kettering Cancer Center, New York, NY 10065 USA; 3 Department of Radiation Oncology, University of Miami Miller School of Medicine, Miami, FL 33136 USA; 4 STTARR Innovation Centre, Radiation Medicine Program, Princess Margaret Cancer Centre, Toronto, Ontario Canada; 5 Department of Medical Biophysics, University of Toronto and Princess Margaret Cancer Centre, Toronto, Ontario Canada; 6 Department of Biomedical Engineering, Ulsan National Institute of Science and Technology, Ulsan, South Korea; 7 Department of Radiology, Memorial Sloan Kettering Cancer Center, New York, NY 10065 USA; 8 Cancer Imaging and Metabolism, Moffitt Cancer Center, Tampa, FL 33612 USA; 9 Department of Radiology, University of Miami Miller School of Medicine, Miami, FL 33136 USA; 10 Molecular Imaging Program, National Cancer Institute, Bethesda, MD 20892 USA; 11 Present Address: Department of Radiology, Perelman School of Medicine, University of Pennsylvania, Philadelphia, PA 19104 USA

## Abstract

Tumor heterogeneity can be elucidated by mapping subregions of the lesion with differential imaging characteristics, called habitats. Dynamic Contrast Enhanced (DCE-)MRI can depict the tumor microenvironments by identifying areas with variable perfusion and vascular permeability, since individual tumor habitats vary in the rate and magnitude of the contrast uptake and washout. Of particular interest is identifying areas of hypoxia, characterized by inadequate perfusion and hyper-permeable vasculature. An automatic procedure for delineation of tumor habitats from DCE-MRI was developed as a two-part process involving: (1) statistical testing in order to determine the number of the underlying habitats; and (2) an unsupervised pattern recognition technique to recover the temporal contrast patterns and locations of the associated habitats. The technique is examined on simulated data and DCE-MRI, obtained from prostate and brain pre-clinical cancer models, as well as clinical data from sarcoma and prostate cancer patients. The procedure successfully identified habitats previously associated with well-perfused, hypoxic and/or necrotic tumor compartments. Given the association of tumor hypoxia with more aggressive tumor phenotypes, the obtained *in vivo* information could impact management of cancer patients considerably.

## Introduction

Cancer is the second leading cause of death in the U.S., with over 1.6 million new cases and half a million deaths each year^1^. Given its prevalence, research has focused on new treatments and an expanded understanding of the nature of the disease. Tumors are commonly heterogeneous^2–5^, stemming from selection pressures on different cancer cell clones^5^. This heterogeneity is largely held to be responsible for the variable outcomes among patients receiving the same therapy and the loss of effectiveness of an agent in a single patient over time^2, 6^. Tumor heterogeneity can be elucidated by mapping subregions of the lesion with differential imaging characteristics, called habitats^7–9^. Delineating tumor habitats *in vivo* is important for determining prognosis and providing effective treatment. Simply detecting tumors may not be enough; the full degree of tissue heterogeneity must also be understood. In this regard, tissue biopsies have the limitation of sampling only a small fraction of the tumor^10, 11^. Therefore, volumetric imaging methods hold promise as a means of characterizing tumor heterogeneity.

Hypoxia is a key determinant of tumor habitats as it favors molecular pathways towards tumor aggressiveness. Hypoxic tumors, often associated with a more aggressive tumor phenotype, are more resistant to chemo- or radiation therapy than well-vascularized, well-oxygenated tumors^12–15^. Hypoxia occurs in solid tumors when the consumption of oxygen exceeds its delivery by the vascular system. In most large clinical studies, the outcome is worse for patients with hypoxic tumors, consistent with the long-held idea that hypoxia confers both chemotherapy and radiation resistance^13–15^. Unexpectedly, higher proportions of hypoxic tumor areas were also found to predict metastasis development in patients treated with surgery alone, pointing to biological effects beyond those linked to drug and radiation resistance^16, 17^.

Magnetic Resonance Imaging (MRI) provides a promising glimpse into tumor heterogeneity. The imaging habitats, comprising the tumor, defined by their distinct physiology can be characterized by their MRI features^7^. In particular, Dynamic Contrast Enhanced (DCE-) MRI can characterize the microenvironment in solid tumors by determining areas of inadequate or heterogeneous perfusion with hyper-permeable vasculature^18^. As tumor oxygenation is dependent on the microcirculation, the technique may be useful to identify tumor areas with poor blood flow, and therefore, hypoxic microenvironments^19^. DCE-MRI has been shown to correlate with Eppendorf O_2_ electrode measurements in cancers^20^. The individual tumor habitats vary in their rate and magnitude of contrast uptake and washout^21^. Discriminating between these MR signals-*versus*-time patterns *in vivo* using MRI is challenging considering: (*i*) the pixel size (100's of microns) is at least an order of magnitude larger than the physical dimensions of the tumor microenvironment (10's of microns); as a result, the detected MR signal reflects an average of different signal-*versus*-time patterns, which is (*ii*) further degraded by noise, both intrinsic, and related to motion and other artifacts. Nonetheless, areas of tumor hypoxia can be detected using the signal-*versus*-time curves of DCE-MRI data as a surrogate marker^21^. The technique is based on an unsupervised pattern recognition (PR) technique that determines the differential signal-*versus*-time curve pattern associated with any given tumor habitat. Previously, the *number* of temporal contrast patterns were determined visually based on the Principal Components (PCs) of the DCE-MRI signal-*versus*-time curves^21^.

Here, a statistical approach for determining the number of significant PCs is presented. Further, this number is utilized in an unsupervised PR algorithm in order to delineate and quantitatively characterize tumor habitats. The technique is examined on simulated data and DCE-MRI data obtained from prostate and brain cancer pre-clinical models, as well as clinical data from patients with sarcoma and prostate cancer.

## Results

### Simulated Data

The goal of the proposed DCE-MRI analysis is to estimate: first, the *number* of habitats (*k*) in the Volume of Interest (VOI); and second, to *determine* the spatial distribution and characteristic signal-*versus*-time curves for each habitat. The procedure is evaluated on several sets of simulated data.

#### Determination of the Number of Habitats

In Stoyanova *et al*.^21^, the number of independent signal-*versus*-time curves in DCE-MRI data, *k*, was determined via visual inspection of the Principal Components (PCs), following Principal Component Analysis (PCA) of DCE-MRI data from the VOI. *k* was the numbers of signal-related PCs.

**Figure 1:**
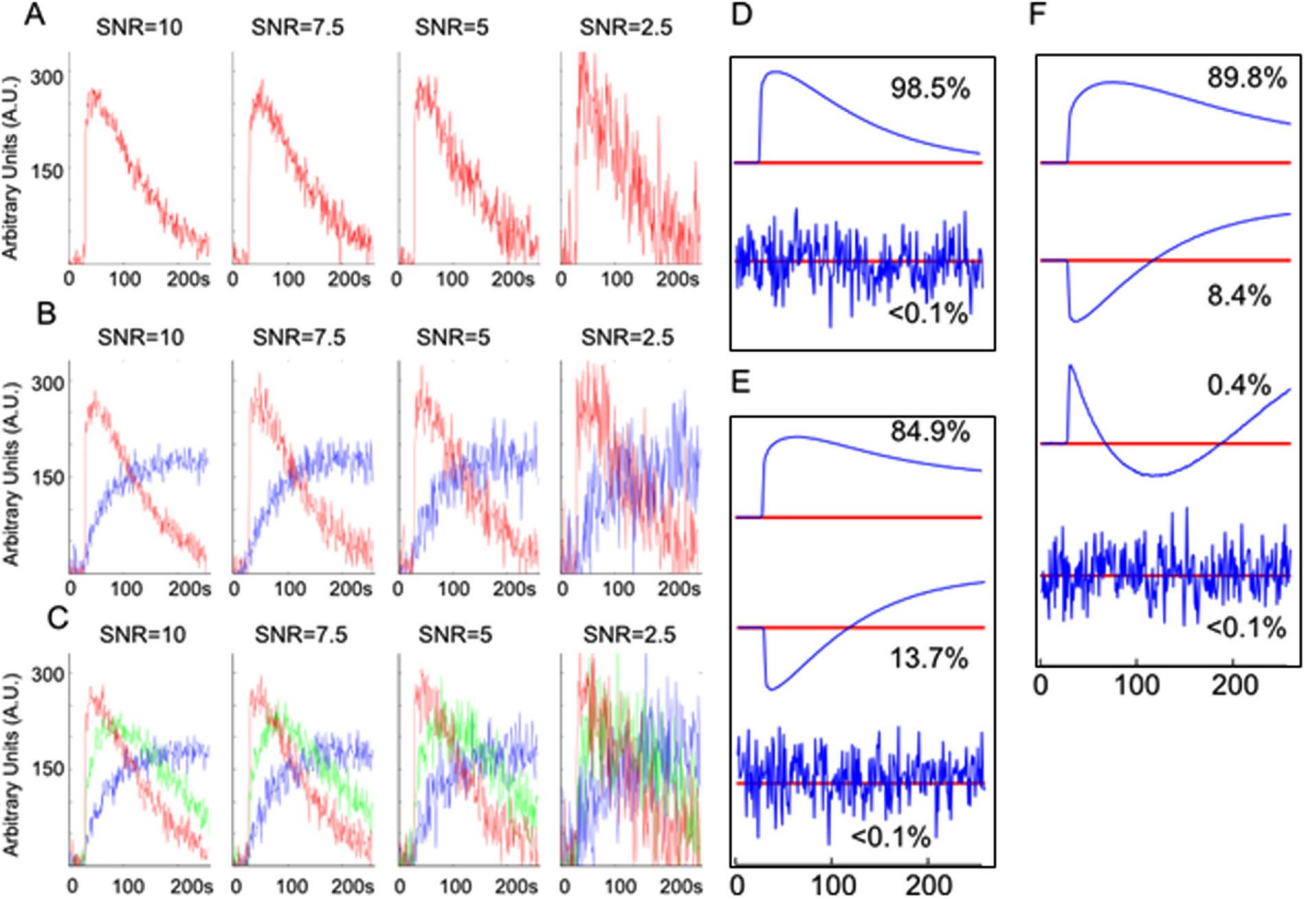
Determination of number of habitats in simulated data. (**A–C**) Representative signal-*versus*-time curves at varying SNR levels for simulated datasets with one, two, and three signal-*versus*-time curve patterns, respectively. (**D**–**F**) Figures show signal-related PCs and the next highest noise-related PC for simulated datasets with one, two, and three signal-*versus*-time curve patterns, respectively. The percent total variance is indicated for each PC.

Here, a statistical procedure for determining *k* is proposed. PCs are ordered by the decreasing amount of signal variance they explain, so the cut-off between signal and noise-related PCs will be the sought number *k*. For illustration in Fig. 1 the PCs of three simulated 2D DCE-MRI datasets are presented. PCA is carried out on the matrixes *D* ^*1*^(*r*, *t*), *D* ^*2*^(*r*, *t*), *D* ^*3*^(*r*, *t*), *r* = 100 × 100, *t* = 1, …, 256. *D* ^*1*^(*r*, *t*) contains a single signal-*versus*-time curve; *D* ^*2*^(*r*, *t*) and *D* ^*3*^(*r*, *t*) contain a mixture of two and three signal-*versus*-time curves, respectively. All signals are simulated with varying amplitudes in the presence of noise (see Methods). Upon visual inspection of the PCs in Fig. 1, it is clear that the PCA analysis of *D* ^*1*^(*r*, *t*), *D* ^*2*^(*r*, *t*) and *D* ^*3*^(*r*, *t*) results in 1, 2 and 3 signal-related PCs, respectively; the remaining, higher order PCs are noise-related. The developed procedure identifies the noise-related PCs as follows:

(i) In the signal-related PCs, the distribution of the first *m* points (pre-contrast) will be different from the distribution of the remaining *N*-*m* points. For each PC, an F-test for variance was performed between the *m* and *N*-*m* points with a p-value threshold of 0.05. The number *k*′ of consecutive PCs that satisfied the F-test was determined.
(ii) The data in the noise-related PCs can be assumed to be normally distributed. The Shapiro-Wilk test will be insignificant in this case (p > 0.05) and vice versa; p < 0.05 for signal related PCs. Again, the Shapiro-Wilk test is applied to the PCs and the number *k*″ of consecutive PCs that failed the Shapiro-Wilk test was determined.
(iii) *k* is determined as *k* = mim(*k*′, *k*″).

To test the performance of the procedure, 4 sets of Gaussian-distributed noise with mean of 0 and variable standard deviation was added to *D* ^*1*^(*r*, *t*), *D* ^*2*^(*r*, *t*) and *D* ^*3*^(*r*, *t*) in order to generate datasets with 2.5, 5, 7.5, and 10 signal-to-noise ratio (SNR) (${\rm{SNR}}=\frac{{\rm{2}}\,{\rm{h}}}{{\rm{\sigma }}}$, where h represents max height of a signal-*versus*-time curve with standard deviation σ^22^). The results of the automatic procedure for identification of the signal-related PCs are shown in Table 1. The determined number was never smaller than the ‘true’ number of shapes *k*, i.e. the procedure never underestimated the number of habitats. Both the F-test and Shapiro-Wilk test performed comparably (paired t-test, p = 0.95). On average, the F-test and Shapiro-Wilk test estimated the correct number of shapes (*k*) in 95.13% and 94.32% of trials (total of 1000), respectively. The F-test and Shapiro-Wilk test overestimated *k* by one (*k* + *1*) in an average of 4.69% and 5.30% of trials and overestimated *k* by 2 or greater (≥*k* + *2*) in an average of 0.15% and 0.37% of trials. Remarkably, when both tests were combined, results were more accurate than from the individual tests. In particular, combining results from both tests estimated the correct number of shapes in 99.60% of trials, overestimated *k* by one in 0.40% of trials, and overestimated *k* by 2 or greater in none of the trials, supporting the use of both tests in the determination of *k*.

**Table 1:** The results from the simulated datasets with one, two, and three signal-*versus*-time curves are shown below.

# of curves in simulated dataset (“ground truth”)	# of curves determined	% of 1000 trials for F-test (*k*′)				% of 1000 trials for Shapiro-Wilk Test (*k*″)				% of 1000 trials for min(*k*′, *k*″)			
		SNR				SNR				SNR			
		10	7.5	5	2.5	10	7.5	5	2.5	10	7.5	5	2.5
1	1*	94.7	93.9	95.0	95.7	94.4	93.9	94.8	94.3	99.8	99.4	99.6	99.6
	2	5.0	5.8	4.9	4.2	5.4	5.5	4.9	5.3	0.2	0.6	0.4	0.4
	> = 3	0.3	0.0	0.1	0.1	0.2	0.6	0.3	0.4	0.0	0.0	0.0	0.0
2	2*	95.3	94.7	95.6	95.2	95.3	94.7	94.5	93.3	99.6	99.6	99.7	99.5
	3	4.5	5.3	4.2	4.5	4.5	4.9	5	6.1	0.4	0.4	0.3	0.5
	> = 4	0.2	0.0	0.2	0.3	0.2	0.4	0.5	0.6	0.0	0.0	0.0	0.0
3	3*	96	94.5	95.3	95.7	94.2	94.0	94.3	94.2	99.4	99.5	99.8	99.7
	4	4.0	5.4	4.4	4.1	5.5	5.6	5.5	5.4	0.6	0.5	0.2	0.3
	> = 5	0	0.1	0.3	0.2	0.3	0.4	0.2	0.4	0.0	0.0	0.0	0.0

The percent of trials (total of 1000) where the specified number of curves were determined are indicated for the two tests and their combination. Neither the tests nor their combination determined a number of curves less than the number of curves in the dataset.

*Number of curves determined was never less than the corresponding “ground truth” number.

#### Spatial Deconvolution of Habitats

**Figure 2:**
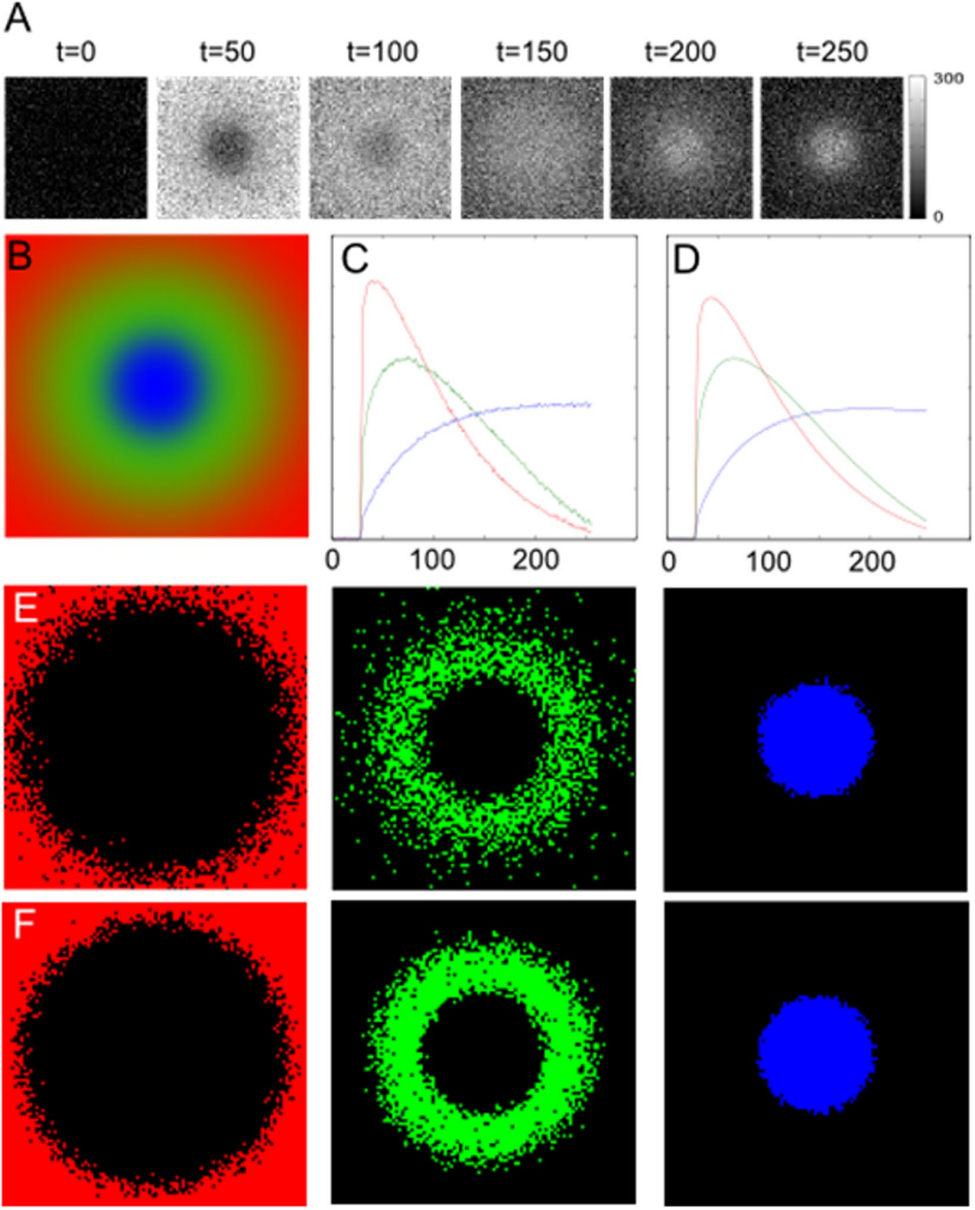
Deconvolution of habitats in a simulated dataset with mixed voxels. (**A**) Representative images of *D* ^*mixed*^(*r*, *t*) (SNR of 2.5) at selected time points (from 0 to 250 s with increments of 50 s); (**B**) Map showing the distribution of weights in each voxel for well-perfused, hypoxic, and necrotic habitats in *D* ^*mixed*^(*r*, *t*), as indicated by the level of red, green, and blue, respectively; (**C**,**D**) Results from cNMF after seeking three solutions in *D* ^*mixed*^(*r*, *t*) at an SNR of 2.5 and 10, respectively. The number of solutions sought was automated through the combined test, which indicated the number of curves present in the dataset; (**E**,**F**) cNMF weights showing the spatial distribution of the recovered well-perfused, hypoxic, and necrotic signal-*versus*-time curves (left to right) for *D* ^*mixed*^(*r*, *t*) at an SNR of 2.5 and 10, respectively.

A dataset *D* ^*mixed*^(*r*, *t*), with three signal-*versus*-time curves was simulated, in which individual voxels contained weighted sums of the three signal-*versus*-time curves. Weights for each signal-*versus*-time curve at each voxel were dependent on the location of the voxel in the dataset. To approximate an idealized distribution of tumor habitats in a DCE-MRI dataset, voxels in the center of the dataset were designated as necrotic tumor areas, voxels in the periphery were designated as well-perfused tumor areas, and voxels in between the center and periphery were set as hypoxic tumor areas (see Methods). Representative images at select time points and a depiction of *D* ^*mixed*^(*r*, *t*) are shown in Fig. 2A,B. Constrained Non-Negative Matrix Factorization (cNMF)^23^, an unsupervised PR algorithm, was applied to *D* ^*mixed*^(*r*, *t*) and the signal-*versus*-time curves for each habitat were readily deconvolved from the dataset using cNMF to find *k* solutions derived from automated determination of the number of habitats (*k* was determined to 3 in *D* ^*mixed*^(*r*, *t*)). Deconvolution was robust to noise, producing similar solutions at an SNR of 2.5 and 10 respectively (Fig. 2B,C). The spatial distribution of each signal-*versus*-time curve, presented by the maps of their corresponding weights recovers successfully the initial distribution of the simulated habitats (Fig. 2D,E).

The approach is implemented in MIM (MIM, Cleveland, Ohio) (see Methods).

### Experimental *in vivo* Data

#### Preclinical Prostate Cancer Model

**Figure 3:**
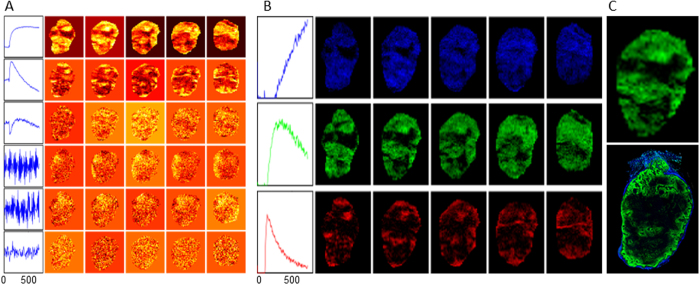
Application of technique for delineation of tumor habitats to a pre-clinical prostate tumor model. (**A**) First six PCs and their corresponding scores from PCA decomposition of DCE-MRI prostate cancer syngeneic tumor dataset. Automatic determination of the number of signal-related PCs revealed three signal-related PCs; (**B**) Signal-*versus*-time curve patterns and their corresponding weights from cNMF analysis of the tumor dataset seeking three unique patterns. Based on the Area Under the Curve (AUC) between 0 and 90 sec of the patterns, the weights are assigned to hypoxic (green), necrotic (blue) and well-perfused (red) habitats; (**C**) Top image shows hypoxic tumor areas in a sample slice, as determined from the present approach (taken from **B**). Bottom image shows hypoxic areas, indicated through pimonidazole staining on a co-registered frozen section for comparison.

The automatic procedure for identification of signal-related PCs and mapping the tumor habitats was validated on a previously analyzed DCE-MRI dataset from a Dunning rat R3327-AT prostate cancer syngeneic hind leg tumor (for experimental and MRI acquisition details, see Methods and Cho *et al*.^18^). Figure 3A shows the PCs and their corresponding scores from PCA decomposition of the tumor dataset up to the first noise-related PC, as determined by the automatic procedure (i.e., the first 6 PCs are shown, demonstrating the noise-related PCs beyond the 3 unique signal-related PCs determined by procedure). Subsequently, the determined number of signal-related PCs was used in cNMF to seek signal-*versus*-time curves for the three unique signal patterns in the tumor dataset. Figure 3B shows the signal-*versus*-time curves derived from cNMF and their corresponding weights in each of the 5 image slices of the tumor dataset. The temporal patterns of the signal-*versus*-time curves bear a strong resemblance to those previously identified^18, 21^ to be associated with well-perfused, hypoxic, and necrotic tumor microenvironments in the dataset. The comparison between the image, depicting the hypoxic environment and the corresponding slice with pimonidazole (a hypoxia marker^24^, displayed in green) staining shows significant areal overlap (Figure 3C). At the same time, there are notable differences between the two maps^18, 21, 25^. Aside from the differences in slice thicknesses (0.79 mm for *in vivo* and 8 µm for *ex vivo*), there are also mechanistic differences. The pimonidazole staining is based on the cellular uptake and metabolism of the 2-nitroimidazole, which depends not only on oxygen level but also on the perfusion (delivery of pimonidazole), cell viability, and cellular nitroreductase activity^24^. Hence, less intense pimonidazole staining is observed in areas farther away from well-perfused areas (red in Fig. 3B). Meanwhile, in the DCE hypoxia map, the intensities are related to the contrast generated from the contrast agent being delivered and leaked (with delayed washout) into the tissue by the vasculature, without entering cells. Spatial heterogeneity of the various habitats beyond hypoxia are represented in the corresponding well-perfused and necrotic maps (Fig. 3B).

#### Preclinical Brain Tumor Model

**Figure 4:**
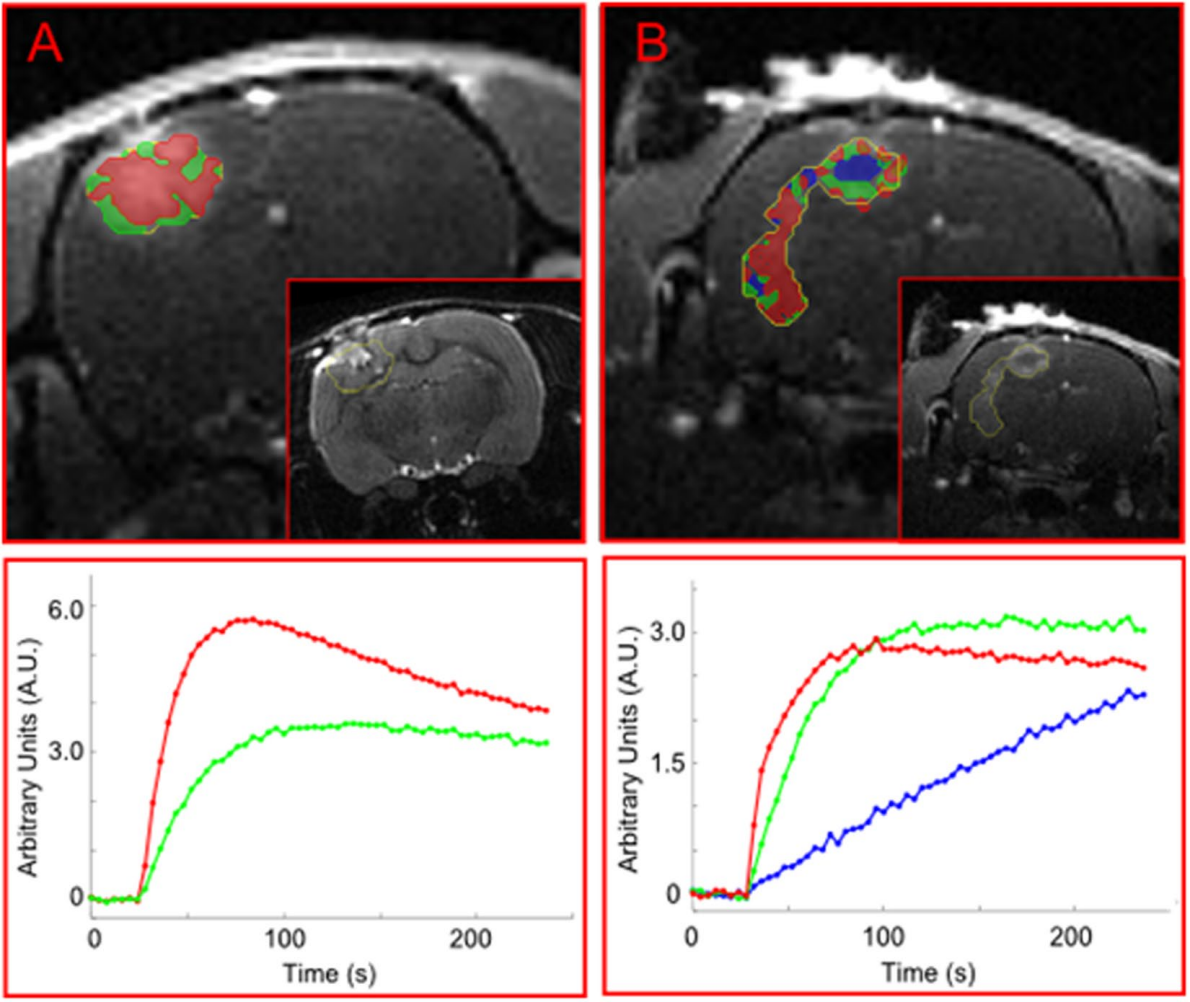
Mapping of tumor habitats in a pre-clinical brain tumor model. (**A**,**B**) Representative coronal slices with mapped tumor habitats in DCE-MRI data from pre-clinical models of brain tumors (left (**A**): U87, right (**B**): RG2). (Insets) Corresponding anatomical images with outlined tumor volume. Automatic determination of the number of signal-related PCs revealed correspondingly two and three signal-related PCs; (Bottom) Corresponding characteristic average signal-*versus*-time curves from each habitat. Red, green, and blue well-perfused, hypoxic, and necrotic habitats respectively.

DCE-MRI data from two preclinical models of brain tumors were analyzed using the procedure: (*i*) U87 tumor, developed from a human Grade IV astrocytoma cell line^26^; and (*ii*) Rat Glioma 2 (RG2) tumor, developed from a malignant, invasive murine glioma cell line^26^. RG2 tumors are more aggressive and rapidly spreading, while U87 tumors are the slower growing of the two types. To achieve equal size at treatment, tumors were allowed to grow for 4–5 weeks and 11–14 days post-implantation for U87 and RG2 tumors, respectively, resulting in similarly sized tumors of volume 0.0279 cm^3^ and 0.0265 cm^3^. Tumors were then treated with Aminolevulinate (ALA)-induced Protoporphyrin IX (PpIX)-mediated Photodynamic Therapy (PDT)^27^. DCE-MRI was performed seven days following PDT, at which time the tumor size of RG2 was markedly increased (0.0623 cm^3^), while U87 remained unchanged (0.0279 cm^3^). The results of the analysis procedure are presented in Fig. 4. Two habitats were identified 7 days post treatment for the U87 tumor and three for the RG2 tumor. In the U87 tumor, 65% of the tumor can be described by fast contrast agent wash-in and wash-out (red), associated with well-perfused^18, 21^ tumor regions (Fig. 3B). The 2^nd^ habitat in the U87 tumor showed delayed contrast wash-in and wash-out, similar to hypoxic areas in previous studies^18, 21, 25, 28^ (Fig. 3B). In the RG2 tumor, the 3 habitats covered 43% (red), 28% (green) and 21% (blue) of the tumor respectively. The 3^rd^ habitat shows contrast agent leakage and diffusion into to tumor areas without functional microvasculature, typically associated with tumor necrosis.

### Clinical *in vivo* Data

#### Sarcoma

**Figure 5:**
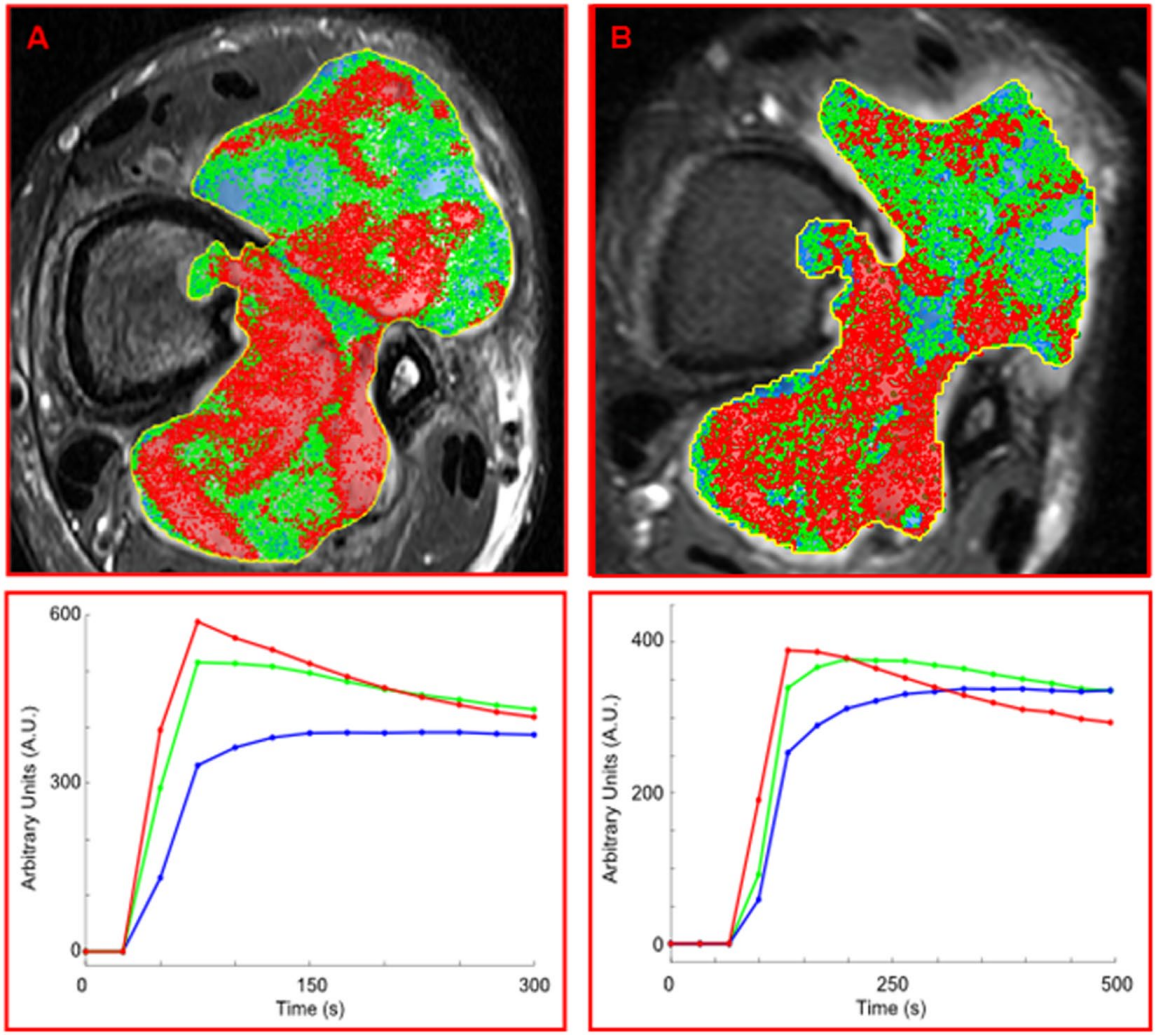
Mapping of tumor habitats in an axial slice of the lower leg of a patient with fibrosarcoma before and after chemotherapy. (**A**) Pre-treatment; and (**B**) After treatment. The post-treatment MRI was aligned with the pre-treatment MRI by rigid fusion. Tumor habitats are presented as follows: ‘well-perfused’ – red; ‘hypoxic’ – green; and ‘necrotic’ – blue. The two imaging exams were 5 months apart. The post-treatment exam is a month after completion of therapy. There are very small changes in the tumor composition, suggesting little therapeutic effect of the chemotherapy. The patient subsequently underwent amputation, with the tumor showing only 10% histologic necrosis. Note that the fraction of the ‘hypoxic/necrotic’ patterns is higher in the anterior part of the tumor while the posterior component is better perfused and harbors infiltrating, viable tumor. (Below) Corresponding average signal-*versus*-time curves from each habitat.

Two sets of DCE-MRI were obtained five months apart from a 38 year-old patient with a grade 2 fibrosarcoma in the lower leg: one pre-chemotherapy and the second a month after completion of treatment (for MRI acquisition details and co-registration, see Methods). The habitat analysis of the two DCE-MRI exams of the patient is shown in Fig. 5. The MRI-estimated tumor volumes were 64 and 50.1 cm^3^, indicating ~20% reduction of the tumor volume between the exams. Given the small number of DCE time-points, the number of tumor habitats could not be determined using the automatic procedure with reliability. However, under the assumption of three habitats, potentially corresponding to well-perfused, hypoxic, and necrotic areas, cNMF was used to explore unique temporal patterns and their locations within the tumor. Remarkably, the recovered signal-*versus*-time curves from both exams resembled those of the simulated well-perfused, hypoxic, and necrotic signal-*versus*-time curves. In addition, the spatial distribution of the patterns was very similar between the two MRI exams, indicating minimal changes in the tumor composition following chemotherapy, and thus, suggesting a lack of a therapeutic effect (Fig. 5). Indeed, the fractions of the well-perfused, hypoxic and necrotic components in the first exam 24%, 21%, and 25% were very similar to the fractions of the same components in the second: 20%, 19%, and 28%. The temporal behavior of the habitats’ signal-*versus*-time curves was also very similar. The well-perfused pattern (red color) was more dominant posteriorly, possibly suggesting the direction of tumor infiltration. Following the second exam, resection of the tumor was performed upon which pathology demonstrated 6.5 × 4.5 × 4.0 mm^3^ mass with 10% necrosis and 90% viable tumor tissue. The estimated tumor volume at resection (49 cm^3^) was very close to the MRI-determined volume (50.1 cm^3^). However, the fraction of the necrotic tissue was overestimated with the habitat analysis. There are various possible explanations for this discrepancy: (*i*) Partial volume contamination from the surrounding muscle, as muscle is characterized with relatively low perfusion^29^ and, due to the relatively large slice thickness in the longitudinal direction (3 mm), voxels on the tumor periphery can be contaminated with signals from the surrounding muscle. Because of the low perfusion of the muscle, this component will have a similar temporal pattern as the necrotic tissue. It can be seen that a large number of voxels on the tumor border, especially anteriorly, are colored as necrotic, which is consistent with the reasoning above. Using the Contract/Expand utility in MIM, the volume of the surface pixels on the tumor was measured to be 3.25 cm^3^, or about 6% of the tumor; (*ii*) Histological slice may not be representative of entire *in vivo* slice due to differences in spatial resolution (in-plane and slice thickness) and quality of slice alignment; (*iii*) As pathologists determine % necrosis histologically at the cellular level on sections from tumor areas that are solid and not grossly necrotic, histological % necrosis may not be representative and may underestimate whole-tumor (or whole-slice) % necrosis determined *in vivo*.

#### Prostate Cancer

**Figure 6:**
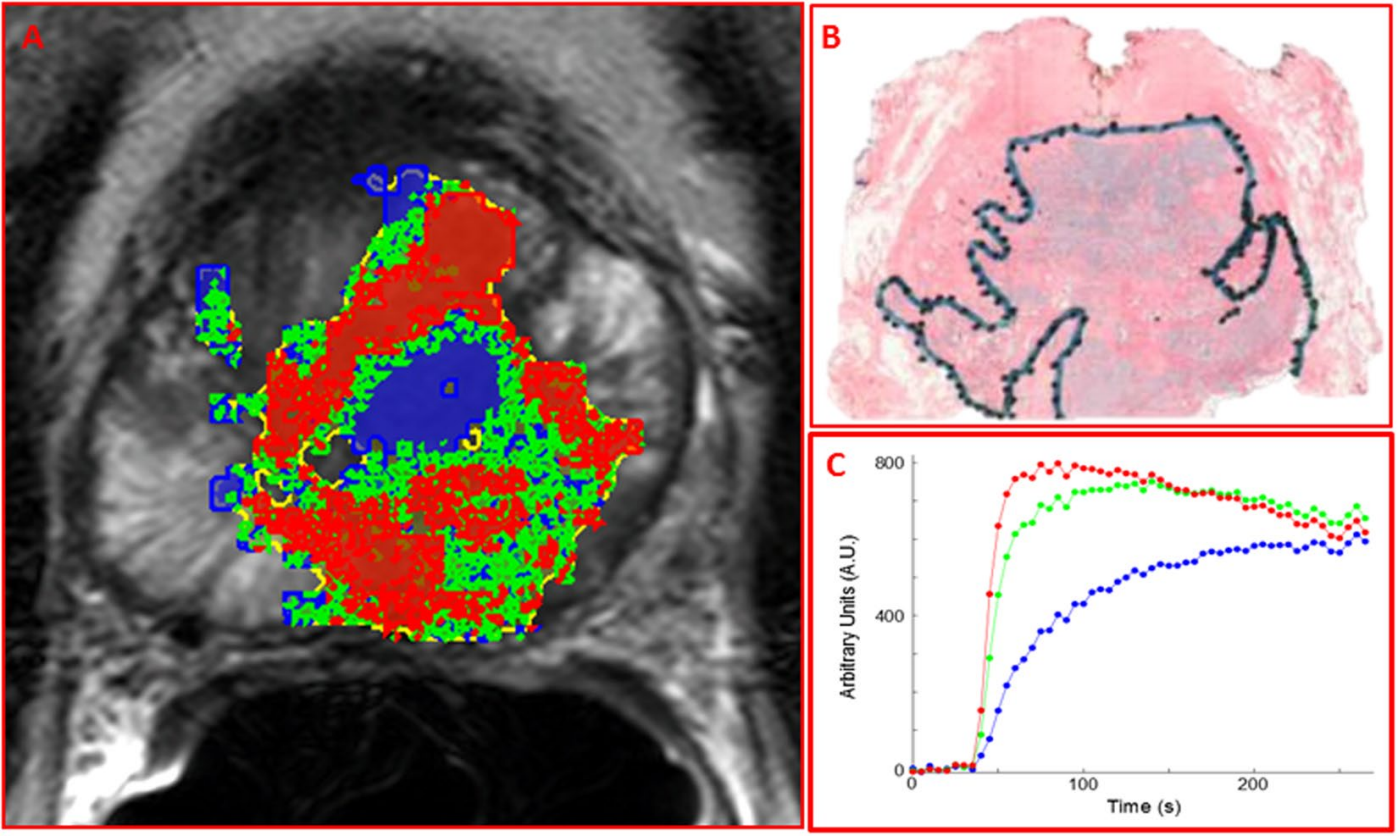
Mapping of tumor habitats in prostate cancer. (**A**) A T_2_-weighted axial MR image slice of the prostate of a patient with prostate cancer. Tumor habitats are presented as follows: ‘well-perfused’ – red; ‘hypoxic’ – green; and ‘necrotic’ – blue; (**B**) Histopathological slide at mid prostate level with outlined tumor area (Gleason Score 9); (**C**) Corresponding average signal-*versus*-time curve from each habitat.

Habitat analysis of the DCE-MRI exam of a patient with prostate cancer is shown in Fig. 6 (for MRI acquisition details, see Methods). The tumor contour was automatically generated by thresholding the Apparent Diffusion Coefficient (ADC) map for values less than 1000 µm/s^2^ ^30–32^. cNMF was applied to the signal-*versus*-time curves from the pixels within that contour. Habitat analysis uncovered three habitats, potentially corresponding to well-perfused, hypoxic, and necrotic areas. Again, the average signal-*versus*-time curves from the recovered habitats resembled closely the simulated ones for well-perfused, hypoxic, and necrotic signal-*versus*-time curves. While necrosis is rarely found in prostate cancer, it is associated exclusively with high-grade tumors (Gleason Score ≥9)^33^. Indeed, histopathological evaluation of the whole-mount step sections with hematoxylin and eosin (H&E) staining confirmed a large prostate Gleason Score 9 tumor, involving almost half of the gland.

## Discussion

An approach for identification of areas of hypoxia in solid tumors from DCE-MRI was previously developed, where the number of differential temporal contrast patterns was inferred via visual inspection of the principal components (PCs) of the dataset^21^. In this paper, a procedure to automate the determination of this number using statistical testing of the PCs is presented. When applied to simulated datasets with varying SNR, the procedure is robust to noise and sensitive to the presence of unique signal-*versus*-time curves. The routine never underestimated the true number of unique signal-*versus*-time curves, suggesting the procedure can be reliably utilized without missing unique tumor habitats in a dataset. The SNR in the *in vivo* data was within the range of the SNR of the simulated datasets. The sensitivity of the procedure was further determined to be at a subpixel level through application on a simulated dataset with voxels containing mixtures of three signal-*versus*-time curves characteristic of well-perfused, hypoxic, and necrotic tumor habitats. The presence of all three signal-*versus*-time curves was detected, and through cNMF, the temporal pattern and spatial distribution of the three signal-*versus* time curve shapes was recovered with good fidelity. In other words, the identity and location of the tumor habitats were recovered from the dataset, despite being below the resolution of the DCE-MRI dataset. Given MRI’s spatial resolution, the presence of different habitats within single voxels can be difficult to resolve by visual inspection, but it can be readily deconvolved through an unsupervised PR technique. In other words, the approach is not only capable of identifying voxels that predominantly follow a given pattern, but also can determine voxels with significant contributions from more than one pattern, effectively increasing the spatial resolution of the data.

The procedure to extract temporal contrast patterns in DCE-MRI datasets can be applied to experimental and clinical data as demonstrated in Figs 3–6. In expectation of further application of the procedure, several potential pitfalls should be noted. First, the procedure involves the use of the Shapiro-Wilk test, a test for normality, based on the assumption that the distribution of the intensities in noisy PCs follows a normal distribution. In experimental datasets, the assumption of normality may not be valid; however, the procedure is not solely reliant on the Shapiro-Wilk test. In addition, an F-test to compare the variance in PCs between data before and after contrast washin is carried out. Noisy PCs with non-normal distributions would tend to increase the number of temporal contrast patterns detected by the Shapiro-Wilk test, but the procedure would account for such an increase, since it uses the minimum number of habitats detected by either the F-test or Shapiro-Wilk test. A sufficient number of time points are required before and after contrast washin in order to perform the F-test in a reliably. For experimental datasets in pre-clinical models, higher temporal resolution in the DCE-MRI can be readily accomplished, but for clinical datasets in patients, this may not be always be possible.

The selection of the ‘purity’ parameter β allows for selecting thresholds addressing a particular problem. If the purpose of the study is to identify strictly the ‘pure’ habitats, β can be set to ≥60%. If analyzing multiple datasets from the same experiments, β should be kept constant to facilitate comparison between the datasets.

The habitats, identified in the pre-clinical and clinical prostate cancer data demonstrate the ability of the technique to identify and quantify the fraction of each habitat in the tumor. While the identified habitats are characterized by contrast agent uptake behavior similar to those found preclinically, their interpretation needs to be validated by additional *ex vivo* or *in vivo* imaging studies. If validated and given the correlation between tumor hypoxia and treatment outcomes, such results could then be used for developing predictive models and/or stratifying patients. The analysis of the prostate cancer patient data indicates the existence of a core with very restricted perfusion. Unfortunately, this result cannot be validated *ex vivo*, since it requires additional staining with hypoxia markers. A larger study for confirming the association of hypoxic/necrosis areas as identified by DCE-MRI with histopathology is underway. Potentially,^18^F-Fmiso PET may be used to relate the accumulation of ^18^F-Fmisonidazole (^18^F-Fmiso), a clinically utilized hypoxia marker^34^, to relate with the hypoxic areas identified by our proposed PR method. Other MR-based methods, beyond DCE-MRI, to assess tissue oxygenation noninvasively *in vivo* include blood oxygen level dependent (BOLD), tissue oxygen level dependent (TOLD) and oxygen enhanced (OE) MRI as well as ^19^F oximetry^15, 35–40^. The required administration of a ^19^F tracer in ^19^F oximetry where the oxygen-level-dependent T_1_ relaxation time of the tracer can be converted to an oxygen concentration by a calibration curve, together with the often restricted ^19^F MR capabilities on clinical MR scanners, limits its clinical applicability, while being a useful preclinical research tool^41^. Easier accessible on clinical scanners are BOLD, TOLD, and OE MRI or combinations thereof which make use of preinstalled MR pulse sequences and do not require special hardware^42, 43^, such as e.g. a ^19^F channel for ^19^F MR oximetry. In BOLD MRI, the contrast is derived from the effective transverse relaxation rate R_2_* (1/T_2_*) measurements, which are sensitive to endogenous paramagnetic deoxyhemoglobin in the blood^37, 38^. In TOLD, the contrast is derived from longitudinal (or spin-lattice) relaxation rate R_1_ (1/T_1_) which is sensitive to the concentration of molecular oxygen in blood plasma and interstitial fluid^37^, while OE MRI measure ΔR_1_ in response to an oxygen breathing challenge^35, 36^. Recently, brain oxygenation was assessed in patients using a multiparametric quantitative BOLD (mqBOLD) approach^43^. In this study, arterial spin labeling (ASL), dynamic susceptibility contrast (DSC) perfusion-weighted MRI, and quantitative BOLD (acquisition of T_2_* and T_2_ maps) were combined to improve the determination of hemodynamic and oxygenation parameters^43^. While the mpBOLD approach is promising to assess the oxygen saturation clinically, the required multiple measurements prolong acquisition time and may propagate errors^43^. Further, R_2_* is not only influenced by deoxygenated hemoglobin but also dependent on tissue iron deposition, calcifications, hematocrit level, extent and structure of the vasculature, and is impacted by tissue interface inhomogeneities of the B_0_ magnetic field^44–47^. Additionally, at higher magnetic fields, more prevalent B_1_ inhomogeneities and shortened T_2_* may degrade image quality and lower the spatial resolution^48^.

Using already widely applied DCE-MRI to evaluate vascular parameters and extract microenvironmental features by the proposed pattern recognitions approach would segue speedy clinical application.

Another application of the technique is to assess treatment response. The habitats identified in the pre-clinical brain tumor data and clinical sarcoma data indicate the possibility of evaluating the spatial response of tumors following treatment. For instance, U87 showed two habitats following PDT, one of which is indicative of a well-perfused established tumor. The interpretation of the second habitat is hindered by the lack of pre-treatment DCE-MRI data or *ex vivo* data. The first would indicate vascular changes in response to treatment, while the latter would allow the *ex vivo* validation of the habitat interpretation. One possibility for the expanding outer rim (green areas) is a compromised blood brain barrier. Another possible interpretation is that as hypoxia is the balance between oxygen delivery and oxygen consumption, and DCE-MRI measure the delivery and washout of Gd-DPTA, though delivery of the CA is compromised as e.g. the result of infiltrating tumor into the surrounding brain, the tissue may or may not be severely hypoxic as of yet. On the other hand, having hypoxic areas on the outside of the tumor could also be the result of the delivery of effective treatment predominantly to the outside of the tumor. The more aggressively growing outer rim is more effective in the synthesis and retention of the PDT sensitizer PpIX from the administered precursor ALA^49, 50^. Finally, similarly to the sarcoma example, the green pixels in the outer rim can be a mixture of tumor and ‘healthy’ brain, resulting in “slowed down” well-perfused pattern. The RG2 tumor presents with a presumably avascular or necrotic core which may be due to the PDT treatment or due to the growth and development of the tumor. Similarly, in the sarcoma example, the (lack of) response to treatment can be identified, and neoadjuvant therapy modified accordingly.

Given the association between tumor hypoxia and cancer treatment outcome/response, approaches to identify tumor habitats have potentially a significant impact on the treatment of cancer patients. The present paper introduces an automatic procedure for delineation of tumor habitats that can be used in conjunction with unsupervised PR techniques to automate the process of determination of tumor habitats from DCE-MRI datasets. By automating the process, the presence of hypoxic tumors potentially can be identified with greater accuracy and sensitivity, as well as higher throughput, allowing for the assignment of patients with hypoxic tumors to more suitable treatments.

## Methods

### Theory

In ideal noise-free scenario let ***s***_*i*_(*t*) be the signal-*versus*-time curve in a voxel *i*, *i* = 1, …, *N* where *N* is the total number of voxels in VOI and *t* is time, *t* = 1, …, *n*. Assuming that ***s***_*i*_(*t*) is a *mixture* of signals from *k* habitats with characteristic signal-*versus*-time curve ***f***_*j*_(*t*), *j* = 1, …, *k*, ***s***_*i*_(*t*) can be represented as the weighted sum of these shapes:

1 $${{\boldsymbol{s}}}_{i}(t)=\sum _{j=1}^{k}\,{{\boldsymbol{A}}}_{j}{{\boldsymbol{f}}}_{j}(t)$$

where ***A***_*j*_ is the amplitude of the *j* ^*th*^ signal-*versus*-time curve ***f***_*j*_(*t*). Let ${\bar{{\boldsymbol{s}}}}_{i}(t)$ be the acquired DCE-MRI signal:

2 $${\bar{{\boldsymbol{s}}}}_{i}(t)={{\boldsymbol{s}}}_{i}(t)+\epsilon $$

where $\epsilon$ is Gaussian noise with mean 0 and standard deviation *σ*, i.e. ${\boldsymbol{\epsilon }}\,\in \,N(0,{\sigma }^{2})$. A 3D DCE-MRI dataset can be organized in a two dimensional matrix $\bar{{\boldsymbol{S}}}(r,t)$ with *r* and *t* being the spatial and time domains, respectively. Assuming ***S***(*r*, *t*) to be the corresponding ‘noiseless’ matrix with ***s***_*i*_(*t*) in its rows, than:

3 $$\bar{{\boldsymbol{S}}}(r,t)={\boldsymbol{S}}(r,t)+{\boldsymbol{\epsilon }}$$

### Determination of Number of Habitats

The rank of the matrix $\bar{{\boldsymbol{S}}}(r,t)$ is *k*, the number of independent DCE components in the dataset, i.e. the number of habitats. Consequently, the number of significant PCs of the PCA decomposition of $\bar{{\boldsymbol{S}}}(r,t)$:

4 $$\bar{{\boldsymbol{S}}}(r,t)={\boldsymbol{A}}(r,t)\cdot {\boldsymbol{P}}(t,t)$$

is equal to *k*. ***A***(*r*, *t*) is the matrix of PC scores (amplitudes) and ***P***(*t*, *t*) is the PCs matrix.

In the case of *k* tumor habitats, resulting in *k* differential signal-*versus*-time curves, PCA of $\bar{{\boldsymbol{S}}}(r,t)$ will yield *k* signal-related PCs.

5 $$\bar{{\boldsymbol{S}}}(r,t)={\boldsymbol{A}}(r,k)\cdot {\boldsymbol{P}}(k,t)+{\boldsymbol{A}}(r,t-k)\cdot {\boldsymbol{P}}(t-k,t)$$

The rest of the PCs will be noise related and can be ignored. $\bar{{\boldsymbol{S}}}(r,t)$ can be represented by the first *k* PCs without statistically significant loss of information, i.e.:

6 $$\bar{{\boldsymbol{S}}}(r,t)\approx {\boldsymbol{A}}(r,k)\cdot {\boldsymbol{P}}(k,t)$$

Two tests are applied to determine *k*:

(i) In the signal-related PCs the distribution of the first *m* points (pre-contrast) will be different from the distribution of the remaining *N*-*m* points.
(ii) The data in the noise-related PCs can be assumed to be normally distributed.

The first assumption is tested by subjecting each PC to an F-test for variance, performed between the *m* and *N*-*m* points with a p-value threshold of 0.05. The number *k*′ of consecutive PCs that satisfy the F-test is determined. The second assumption is evaluated through application of the Shapiro-Wilk test to each PC, supposing that the test will be significant for signal-related PCs (p < 0.05) and vice versa (p > 0.05) for noise-related PCs. The number *k*″ of consecutive PCs that failed the Shapiro-Wilk test is determined. Finally, *k* is estimated as *k* = min(*k*′, *k*″).

### Pattern Recognition (PR)

Constrained Non-Negative Matrix Factorization (cNMF)^23^, an unsupervised PR algorithm, is applied to the data matrix $\bar{{\boldsymbol{S}}}(r,t)$, seeking *k* solutions of basic temporal curves ***F***(*k*, *t*) and their weights ***W***(*r*, *k*):

7 $$\bar{{\boldsymbol{S}}}(r,t)\approx {\boldsymbol{W}}(r,k)\cdot {\boldsymbol{F}}(k,t)$$

The goal is to recover ***A***_*j*_ and ***f***_*j*_(*t*) [Eq. 1] and the rationale of the approach is that ***f***_*j*_(*t*)’s are in the rows of ***F***(*k*, *t*) and ***A***_*j*_’s are in the rows of ***W***(*r*, *k*).

cNMF assumes that each image in the DCE-MRI series represents *k* tissue types with individually associated basic signal-*versus*-time curves. In other words, cNMF seeks a representation of $\bar{{\boldsymbol{S}}}(r,t)$ as the sum of the product of *k* basic contrast signatures ***F***(*k*, *t*) and their weights ***W***(*r*, *k*). Assuming that *k* = 3 and for a given voxel ***A***_1_ is the weight of the well-perfused pattern ***f***_1_(*t*), characterized by rapid contrast uptake followed by rapid washout; ***A***_2_ is the weight of the ‘hypoxic’ pattern ***f***_2_(*t*), characterized by delayed contrast build-up and washout; and ***A***_3_ is the weight of the necrotic pattern ***f***_3_(*t*), which exhibits slow or no contrast uptake and no discernible washout. The fraction of each component in this voxel can be calculated as a percentage of the sum of the three amplitudes (***A***_1_, ***A***_2_ and ***A***_3_). For display and quantitation purposes, however, a rule for assigning voxels to a given habitat may be required. This can be achieved by introducing a threshold β, describing how ‘pure’ a given pattern is in a voxel. For example, a voxel might be composed of 60% well-perfused, 30% hypoxic and 10% necrotic tissue. For β = 50%, this voxel will be assigned to the well-perfused habitat; for β = 30% - the voxel will be assigned to two habitats (well-perfused and hypoxic) and for β = 70% - the voxel will remain unassigned. Therefore, β represents a tradeoff between ‘purity’ and number of pixels assigned: the higher the ‘purity’ requirement, the smaller the number of pixels assigned to a given habitat. In the examples shown in the manuscript, β = 50% was used since on one hand, its is high enough to assure that the dominant pattern in the pixel is quite ‘pure’ and on the other hand the majority of pixels will be assigned.

### Implementation

The analysis pipeline is implemented in MIM (MIM, Cleveland, Ohio). Upon uploading the imaging data, VOI (the tumor) is manually outlined. PCA and cNMF are implemented using Java plugins and applied to signal-*versus*-time curves for all pixels within the VOI^21^. PCs are displayed in separate windows along with the percentage of the variance, associated with each PC. The test for determination of *k* is then carried out. cNMF inputs include *k*, the number of pre-contrast series and β, the threshold for voxel assignment to a specific habitat. As a pre-processing step, each signal-*versus*-time curve is baseline corrected by subtraction of the average intensity from the pre-contrast sets. The resulting cNMF patterns are sorted in descending order based on the area under the curve (AUC) between 0 and 90 sec post contrast agent injection. The corresponding weights are sorted accordingly and mapped over the VOI, given that the % of the pattern is ≥β.

### Simulated Datasets

A dataset was simulated to represent a 2D DCE-MRI acquired on a 100 × 100 grid. Three functional forms were used to simulate ***f***_*j*_(*t*) corresponding to: (***i***) well-perfused tumor areas, characterized by rapid contrast uptake followed by rapid washout; (***ii***) hypoxic areas, which are regions of reduced vascularization, associated with delayed contrast build-up and washout; and (***iii***) necrotic areas, which exhibit slow or no contrast uptake and no discernible washout. The ‘extended’ Tofts model^51, 52^ was used to generate ***f***_*j*_(*t*) using the following pharmacokinetic constants: K^trans^ (related to perfusion and permeability per unit volume of tissue): 0.3, 0.1, and 0.03 min^−1^ and k_ep_ (rate constant between Extracellular Extravascular Space (EEC) and plasma): 0.8, 0.2, and 0.01 min^−1^. A synthetic Parker fixed population average was used as the Arterial Input Function (AIF)^53^ in this model.

Three basic datasets *D* ^*1*^(*r*, *t*), *D* ^*2*^(*r*, *t*), and *D* ^*3*^(*r*, *t*) were simulated, containing one, two and three of the signal-*versus*-time curves described above. To test the performance of the procedure, 4 sets of Gaussian-distributed noise with a mean of 0 and variable standard deviation was added to generate datasets with 2.5, 5, 7.5, and 10 signal-to-noise ratio (SNR) (${\rm{S}}{\rm{N}}{\rm{R}}=\frac{2\,{\rm{h}}}{\sigma }$, where h is the max height of a signal-*versus*-time curve with standard deviation σ). The curves in the three datasets are shown in Fig. 1A.

A fourth set, *D* ^*mixed*^(*r*, *t*), with three signal-*versus*-time curves was simulated, in which individual voxels contained weighted sums of the three signal-*versus*-time curves. Weights for each signal-*versus*-time curve at each voxel were dependent on the location of the voxel in the dataset. To approximate an idealized distribution of tumor habitats in a DCE-MRI dataset, voxels in the center of the dataset were designated as necrotic tumor areas, voxels in the periphery were designated as well-perfused tumor areas, and voxels in between the center and periphery were set as hypoxic tumor areas. Consequently, weights for the necrotic signal-*versus*-time curve were generated using a 2D Gaussian distribution, *G*(*c*, 10, 1), with a center at the midpoint of the image, *c*, standard deviation of 10 voxels, and peak value of 1; weights for the well-perfused signal-*versus*-time curve were generated through an “inverted” 2D Gaussian distribution,*W*(*c*, 45), where *W*(*μ*, *σ*) = 1 − *G*(*μ*, *σ*, 1), and µ and σ represent the center and standard deviation of the 2D Gaussian distribution described by *G*; and finally, weights for the hypoxic signal-*versus*-time curve were created using an “inverted” combination of the prior two distributions, *H*(*c*, 15, 25), where *H*(*μ*, *σ*_1_, *σ*_2_) = 1 − (G(*μ*, *σ*_1_, 1) + *W*(*μ*, *σ*_2_)). Weights for each voxel were then normalized such that the weights for all signal-*versus*-time curves patterns in a single voxel added to 1. Representative images at select time points and a depiction of *D* ^*mixed*^(*r*, *t*) are shown in Fig. 2A,B.

### Experimental *in vivo* Data

#### Preclinical Prostate Cancer Model

Animal studies were conducted in compliance with protocols approved by the Institutional Animal Care and Use Committee of Memorial Sloan-Kettering Cancer Center (MSKCC). The acquisition of DCE-MRI data and tumor micronenvironmental characteristics from a Dunning rat R3327-AT prostate cancer syngeneic tumor was described in detail previously^18^. Briefly, the tumor was implanted subcutaneously on the right hind leg of a Copenhagen rat. DCE-MRI was acquired at 5.347 s temporal resolution for ~2 min prior to Gadolinium-Diethylene-Triamine-Pentaacetic Acid (Gd-DTPA) (Magnevist, Bayer AG, Leverkusen, DE) injection, followed by ~20-min dynamic acquisition, resulting in 256 image sets (5 tumor slices each). The voxel size was 0.273 × 0.273 × 0.79 mm^3^ (0.059 mm^3^), 128 × 128 in-plane matrix, 35 × 35 mm^2^ field-of-view, repetition time (TR) = 41.775 ms, echo time (TE) = 3.1 ms, flip angle = 30°. The average SNR within the VOI was approximately 7.5. The excised tumor section was stained for pimonidazole, a hypoxia marker injected 1 h prior to tumor excision^14, 18^

#### Preclinical Brain Tumor Model

Animal studies were conducted in compliance with protocols approved by the Animal Care Committee (ACC) of University Health Network, Toronto, Canada. Two preclinical models of brain tumors were analyzed: (*i*) a U87^26^ tumor, developed from a human Grade IV astrocytoma cell line presenting many of the classical molecular hallmarks of advanced human glioma disease, specifically increased EGFR expression. Tumors were grown in immunocompromised Rag2/SCID rats (SD-Rag2^tm1sage^, SageLabs, Boyertown, PA, USA) and develop with a highly vascular morphology. 250,000 U87 cells, suspended in Neurobasal-A (Life Technologies, Carlsbad, CA, USA), were injected 3 mm deep into the neocortex of 14–16 week old female rats; and (*ii*) a Rat Glioma 2 (RG2)^27^ tumor, developed from malignant, invasive murine glioma cell lines. 5000 RG2 cells, suspended in Hank’s Balanced Salt Solution (HBSS, Life Technologies, Carlsbad, CA, USA), were injected 3 mm deep into the neocortex of 16–18 week old female Fischer (CDF) Rats (Charles River Laboratories, Wilmington, MA, USA). In both preclinical models, cells were injected 3 mm to the left of the midline and 3 mm above the bregma using a sterile Hamilton Neuros Syringe with a stereotactic frame. Tumors were allowed to grow for 4–5 weeks and 11–14 days post-implantation for U87 and RG2 tumors, respectively, resulting in similar-sized tumors, and at which time ALA-induced PpIX Photodynamic Therapy (PDT) was delivered to the tumors. The treatment parameters were; administration of 100 mg/kg^−1^ ALA injected i.p. followed 4 h later by delivery of 12J of 635 nm light. The PDT-mediated cytotoxic dose follows a steep spatial gradient, governed by the local ALA uptake and thus PPIX synthesis is dominated by the light scattering and absorption in the brain. Hence, it results in spatially varying tissue responses potentially including necrosis, apoptosis, edema and inflammation. Post-PDT, DCE-MRI was performed 7 days post light exposure to permit resolution of the immediate inflammation. The rat brain tumor MRI was performed on a 7 T Biospec USR 70/30 (Bruker Corporation, Ettlingen, Germany). The imaging protocol consisted of T_2_-weighted MRI, DCE-MRI with matched 25.6 × 25.6 mm fields-of-view and center slices. DCE-MRI involved a 2D-FLASH sequence (TR/TE: 40/2.5 ms, flip angle = 35°, 100 × 100 matrix, 256 µm × 256 µm in-plane resolution, 5 slices, 1 mm slice thickness, 60 repetitions per slice, 4 s temporal resolution, 4 min acquisition time). Five DCE series were acquired prior to contrast agent (Gd-DTPA) injection. The average SNR within the VOI was approximately 12.

### Clinical *in vivo* Data

#### Sarcoma

This retrospective study was conducted under the approval of the Institutional Review Board at the University of Miami; informed consent was exempted. Two sets of DCE-MRI were obtained five months apart from a 38-year old patient with a grade 2 fibrosarcoma in the lower leg: one pre-chemotherapy and the second a month after completion of treatment. The first exam was carried out on a 3T Skyra MR Scanner (Siemens, Erlangen, Germany). DCE-MRI data were acquired using a T_1_ axial vibe fat-saturated sequence (4.1 ms TR, 1.87 ms TE) with a ~2 min acquisition prior to Gd-DTPA injection, followed by ~5 min dynamic acquisition at a temporal resolution of 25 s, resulting in 13 image sets with 52 slices each. The voxel size was 0.521 × 0.521 × 3.5 mm^3^ (0.950 mm^3^), 384 × 384 in-plane matrix, 207 × 207 mm^2^ field-of-view. The average SNR within the VOI was approximately 6. The second exam was carried out on a 1.5T Symphony MR Scanner (Siemens, Erlangen, Germany). DCE-MRI data were acquired using a T_1_ axial vibe fat-saturated sequence (5.3 ms TR, 2.75 ms TE) with a ~2 min acquisition prior to Gd-DTPA injection, followed by ~7 min dynamic acquisition, at a temporal resolution of 33 s, resulting in 14 image sets (30 slices each). The voxel size was 0.605 × 0.605 × 3.5 mm^3^ (1.281 mm^3^), 256 × 256 in-plane matrix, 155 × 155 mm^2^ field-of-view. The average SNR within the VOI was approximately 3. The two exams were coregistered in MIM by rigid fusion.

#### Prostate Cancer

Prostate MRI data was obtained from The Cancer Imaging Archive (TCIA)^54^. Imaging studies were performed on a 3T Achieva (Philips Medical Systems, Best, Netherlands). Details about coils and patient preparation are given elsewhere^55^. The imaging protocol included triplanar T_2_-weighted turbo spin-echo MR imaging, axial unenhanced T_1_-weighted MRI and axial 3D fast field-echo DCE-MRI. Axial DCE-MRI were obtained before, during, and after a single-dose injection of Gd-DTPA at a dose of 0.1 mmol/kg through a peripheral vein at a rate of 3 mL/s via a mechanical injector (Spectris MR Injection System; Medrad). DCE-MRI data were acquired using a 10-section 3D fast field-echo sequence, with a phase direction from left to right without fat saturation (TR/TE: 5.5/2.1 ms). Four unenhanced sets (13 s total acquisition time prior to Gd-DTPA injection) and approximately 96 contrast-enhanced image sets of images were acquired sequentially without a delay between acquisitions. A total of approximately 1000 images were obtained during DCE-MRI. The voxel size was 0.86 × 1.18 × 6 mm^3^ (6.0888 mm^3^), 256 × 256 in-plane matrix, 260 mm^2^ field-of-view. The procedure was applied to VOI, automatically generated by thresholding the Apparent Diffusion Coefficient (ADC) map for values less than 1000 µm/s^2^ ^30–32^. The average SNR within the VOI was approximately 3.

The patient underwent robotic-assisted radical prostatectomy. Following the prostatectomy procedure, the specimen was processed within an MRI-based, patient-specific specimen mold^56^. The specimen was evaluated as whole-mount step section with H&E staining, and the tumor outlined by an experienced genitourinary pathologist with >25 years of experience.
